# Prevention of exacerbation in patients with moderate-to-very severe COPD with the intent to modulate respiratory microbiome: a pilot prospective, multi-center, randomized controlled trial

**DOI:** 10.3389/fmed.2023.1265544

**Published:** 2024-01-05

**Authors:** Jian-lan Hua, Zi-feng Yang, Qi-jian Cheng, Yao-pin Han, Zheng-tu Li, Ran-ran Dai, Bin-feng He, Yi-xing Wu, Jing Zhang

**Affiliations:** ^1^Department of Pulmonary and Critical Care Medicine, Zhongshan Hospital, Shanghai Medical College, Fudan University, Shanghai, China; ^2^State Key Laboratory of Respiratory Disease, National Clinical Research Center for Respiratory Disease, Guangzhou Institute of Respiratory Health, The First Affiliated Hospital of Guangzhou Medical University, Guangzhou Medical University, Guangzhou, China; ^3^Department of Pulmonary and Critical Care Medicine, Ruijin Hospital, Institute of Respiratory Diseases, Shanghai Jiao Tong University School of Medicine, Shanghai, China; ^4^Shanghai Key Laboratory of Lung Inflammation and Injury, Shanghai, China

**Keywords:** chronic obstructive pulmonary disease, vaccination, inhaled antibiotics, probiotic, amikacin

## Abstract

**Introduction:**

Considering the role of bacteria in the onset of acute exacerbation of COPD (AECOPD), we hypothesized that the use of influenza-*Streptococcus pneumoniae* vaccination, oral probiotics or inhaled amikacin could prevent AECOPD.

**Methods:**

In this pilot prospective, muti-central, randomized trial, moderate-to-very severe COPD subjects with a history of moderate-to-severe exacerbations in the previous year were enrolled and assigned in a ratio of 1:1:1:1 into 4 groups. All participants were managed based on the conventional treatment recommended by GOLD 2019 report for 3 months, with three groups receiving additional treatment of inhaled amikacin (0.4 g twice daily, 5–7 days monthly for 3 months), oral probiotic *Lactobacillus rhamnosus* GG (1 tablet daily for 3 months), or influenza-*S. pneumoniae* vaccination. The primary endpoint was time to the next onset of moderate-to-severe AECOPD from enrollment. Secondary endpoints included CAT score, mMRC score, adverse events, and survival in 12 months.

**Results:**

Among all 112 analyzed subjects (101 males, 96 smokers or ex-smokers, mean ± SD age 67.19 ± 7.39 years, FEV_1_ 41.06 ± 16.09% predicted), those who were given dual vaccination (239.7 vs. 198.2 days, *p* = 0.044, 95%CI [0.85, 82.13]) and oral probiotics (248.8 vs. 198.2 days, *p* = 0.017, 95%CI [7.49, 93.59]) had significantly delayed onset of next moderate-to-severe AECOPD than those received conventional treatment only. For subjects with high symptom burden, the exacerbations were significantly delayed in inhaled amikacin group as compared to the conventional treatment group (237.3 vs. 179.1 days, *p* = 0.009, 95%CI [12.40,104.04]). The three interventions seemed to be safe and well tolerated for patient with stable COPD.

**Conclusion:**

The influenza-*S. pneumoniae* vaccine and long-term oral probiotic LGG can significantly delay the next moderate-to-severe AECOPD. Periodically amikacin inhalation seems to work in symptomatic patients. The findings in the current study warrants validation in future studies with microbiome investigation.

**Clinical trial registration:**https://clinicaltrials.gov/, identifier NCT03449459.

## 1 Introduction

Chronic obstructive pulmonary disease (COPD) is preventable and treatable common disease characterized by incompletely reversible and progressive development of airflow limitation (1). It was estimated that there were nearly 300 million patients with COPD worldwide in 2017 (2). COPD has caused at least 5.8% of total deaths worldwide each year and has become the third leading cause of death in 2019 (3). In China, the annual direct medical expenses for COPD were between 72 and 3,565 US dollars *per capita*, which accounts for about 40% of the total income of an ordinary family (4). On average, each COPD patient experiences 0.5 to 3.5 acute exacerbations per year, which is an important reason for the increase in hospitalization, disease progression and mortality as well as the decline in health (5, 6). An epidemiological study in China showed that each patient hospitalized with AECOPD spent about RMB 11,598 in treatment per year (7). Reducing the incidence of AECOPD may help slow the progression of the disease and improve the quality of life of patients (8). Therefore, targeted interventions for patients with stable COPD are particularly important.

Among the adults with a diagnosis of COPD, those who have exacerbation history, greater disease severity, higher symptom burden, significant comorbidities and higher blood eosinophil count are more likely to develop moderate-to-severe exacerbation (9, 10). Management of those modifiable risk factors is of great value for exacerbation prevention. At present, several types of drugs have been proven by different levels of evidence to reduce exacerbation frequency in patients with COPD, mainly including inhaled steroids, long-acting bronchodilators, phosphodiesterase inhibitors and mucolytics (11). Non-pharmacological therapies such as smoking cessation, vaccination, and pulmonary rehabilitation are also recommended for the prevention of AECOPD (12). Yet, 22% ~ 40% of COPD patients still experience at least one moderate or severe exacerbation each year (13). This forces us to reflect on the limitation of current preventive measures and seek new methods to prevent acute exacerbation based on risk factors.

Chronic respiratory bacterial colonization has recently been increasingly noticed to play an important role in the pathogenesis of COPD. Potential pathogenic microorganisms could be isolated from the lower respiratory tract of as many as 74% of COPD patients, which was much higher than that of healthy people (14). Similarly, up to 80% of AECOPD were related to bacteria or viruses (15). Therefore, we believe that improving the microenvironment of the lower respiratory tract may contribute to the prevention of AECOPD. Aminoglycosides conserve good susceptibility to Gram-negative bacteria, but the poor pulmonary penetration and the high frequency of side effects in systemic administration limit their clinical use for years. However, inhaled antibiotics can theoretically compensate for the shortcomings of systemic delivery, thus having a great potential for decolonization in COPD (16). Intermittent use of low-dose macrolide antibiotics, such as azithromycin, was found to reduce the incidence of AECOPD and greatly decrease the total respiratory bacterial load (17, 18). However, macrolides were more likely to act as an inflammation modulator rather than an antimicrobial agent in preventing acute exacerbation (19). Thus, the role of modulating respiratory microbiota in using antibiotic prophylaxis for AECOPD prevention has rarely been studied. Influenza vaccination has been believed to reduce the frequency of AECOPD, the number of outpatient visits, hospitalizations, and mortality (20, 21). Similarly, *Streptococcus pneumoniae* vaccine was associated with the reduced risk of hospitalization for patients with moderate to severe COPD (22, 23). Given that influenza virus and *S. pneumococcal* infections are among the most common microbial causes of AECOPD (10, 14), we wondered if the inoculation of both vaccines simultaneously might be more effective. In addition, Alexandre et al. reported that probiotics might be related to the lower prevalence of respiratory infection and the decline of respiratory colonization, which suggest the potential application of probiotics in COPD management (24, 25). In particular, oral intake of *Lactobacillus rhamnosus* GG has been shown to reduce the adhesion of potential pathogens in respiratory tract and reduce the pulmonary exacerbations of cystic fibrosis in several studies (26–28), which had brought attention to the application of probiotics in COPD management.

Based on the previous evidence, we hypothesized that the preventive use of influenza-*S. pneumoniae* vaccines, oral probiotics or inhaled antibiotics (amikacin) during the stable phase of COPD could contribute to reduced respiratory colonization and improved airway microenvironment, so as to delay the progression of disease and improve life quality. Therefore, an exploratory prospective, randomized controlled trial was done to assess efficacy and safety of the three interventions in preventing AECOPD. This study also aimed at verifying the possible improvement of these preventive measures on the respiratory symptoms of patients with COPD.

## 2 Materials and methods

### 2.1 Trial design

This was a multi-center, parallel, prospective, randomized, controlled trial carried out in Zhongshan Hospital Affiliated to Fudan University, The First Affiliate Hospital of Guangzhou Medical University, and Ruijin Hospital Affiliated to Shanghai Jiaotong University from May 2019 to April 2021. Eligible participants were randomly assigned to conventional treatment group, aerosol inhaled amikacin group, oral probiotic group and vaccination strategy group according to the ratio of 1:1:1:1 through the block random method. The total follow-up period was 12 months after enrollment.

### 2.2 Inclusion criteria

The subjects were enrolled and randomized into the study group if all of the following criteria were met: (1) written informed consent must be obtained before any assessment is performed; (2) male or female adults aged 18–80 years; (3) diagnosed with COPD according to the Global Initiative for Chronic Obstructive Pulmonary Disease 2019 (GOLD 2019) report [The ratio of postbronchodilator (salbutamol 400 μg) forced expiratory volume in 1 s (FEV_1_) to force vital capacity (FVC) <0.70]; (4) moderate-to-very severe airflow limitation (postbronchodilator FEV_1_ < 80% of the predicted value); (5) a documented history of at least two COPD exacerbation in the previous 12 months that required treatment with systemic glucocorticoids and/or antibiotics; (6) in the stable stage of COPD.

### 2.3 Exclusion criteria

Exclusion criteria included: (1) patients who have clinically significant and chronic hepatic, renal and gastrointestinal abnormalities or malignant tumor (except for lung cancer) which could interfere with the assessment of the efficacy and safety of the study treatment; (2) patients who are in critical conditions; (3) patients who have had a COPD exacerbation that required treatment with antibiotics and/or systemic corticosteroids or an acute exacerbation of any other diseases in the 4 weeks prior to screening; (4) patients with concomitant pulmonary disease including, but not limited to, bronchiectasis, interstitial lung disease, asthma; (5) patients who are highly likely to be lost during the three-month treatment and the one-year follow up; (6) pregnant or nursing (lactating) women; (7) patients who have been vaccinated against influenza in the current year, or against *S. pneumoniae* within 5 years, or have vaccination contraindications; (8) patients who are allergic to amikacin or other aminoglycosides; (9) patients who have participated in any interventional clinical trials in the 3 months prior to screening; (10) patients with mental diseases or cognitive disorders which could interfere with treatment and follow-up; (11) patients with long-term use of oral corticosteroids; (12) patients with α-1 antitrypsin deficiency.

### 2.4 Interventions

For patients in conventional treatment group, we prescribed long-acting muscarinic antagonists (LAMA) or long-acting β2 agonists/long-acting muscarinic antagonists (LABA/LAMA) or inhaled corticosteroids/long-acting β2 agonists (ICS/LABA) or LAMA/LABA/ICS according to the individualization of the subjects and GOLD 2019 report (29). Tobacco cessation support was also provided. Subjects in conventional treatment group were given only the conventional therapy without any additional intervention, while subjects in other groups were given additional interventions based on the above-mentioned conventional therapy.

For patients in oral probiotic group, they were additionally given Culturelle^™^ DIGESTIVE HEALTH 30 CT (VCAP) (10 Billion Claim) which consists of 100% *Lactobacillus rhamnosus* GG (LGG),1 tablet, q.d., for 3 months (30–32). For patients in aerosol inhaled amikacin group, they were additionally given 0.4 g Amikacin sulfate injection configured with 5 mL saline in the form of aerosol inhalation intermittently for 3 months (b.i.d., 5–7 days per month). In order to observe and cope with adverse events timely, subjects were admitted to the ward during inhaling nebulized amikacin (16, 33–35). For patients in vaccination strategy group, Influenza Vaccine recommended by World Health Organization (WHO) and imported 23-Valent Pneumococcal Polysaccharide Vaccine approved by China Food and Drug Administration (CFDA) were vaccinated by professional nurses (36–39). The two vaccinations were separated by 3–5 days to avoid overlap of adverse events.

### 2.5 Endpoints

The primary endpoint was the number of days from enrollment to the first moderate-to-severe AECOPD that required treatment with systemic glucocorticoids and/or antibiotics. Secondary endpoints included COPD Assessment Test (CAT) score, modified Medical Research Council (mMRC) Questionnaire, adverse events and survival. Colonization of potential pathogenic bacteria, microbiome and cytokines such as IL-6, IL-8, and IL-10 in induced sputum, as well as serum CRP levels were also planned to be collected for analysis (40). Unfortunately, these outcomes were not successfully measured because the outbreak of coronavirus disease 2019 (COVID-19) made it difficult to collect blood and sputum samples.

### 2.6 Data collection

We followed up each subject for a total of 12 months, including a baseline visit (on the day of enrollment), a 3-month follow-up, a 6-month follow-up, and a 12-month follow-up visit. Subjects were visited on site at baseline and the 3-month follow-up. Due to the COVID-19 pandemic, we had to adopt telephone interviews for the 6-months follow-up and 12-months follow-up visit. For the same reason, considering that the one-year follow-up had been completed, the planned 15-months follow-up visit is regretfully canceled after careful decision.

### 2.7 Statistical analysis

Statistical analyses were performed using SPSS 22.0. Analysis of variance (ANOVA) and Dunnett’s *t*-test were used to compare the primary endpoint (the number of days from enrollment to the first moderate-to-severe AECOPD) of all subjects between the intervention groups and the conventional treatment group. For the secondary endpoints (including CAT score, mMRC score, adverse effects, and survival), we used ANOVA to compare the difference between groups at baseline visit, 3-month follow-up, 6-month follow-up, and 12-month follow-up, respectively. In addition, we divided all subjects into 4 subgroups in terms of whether they were severely or very severely airflow limited (GOLD III or IV), whether they had a high risk of exacerbation, whether they had a high symptom burden (CAT ≥10 and mMRC ≥2) or whether they were labeled GOLD D. Then all subjects were analyzed by self-control paired *t* test within subgroups. *p* value of less than 0.05 was considered statistically significance.

### 2.8 Ethic

The trial has been approved in the Ethics Committee of Zhongshan Hospital of Fudan University (B2017-197R) and registered at Clinical Trials (NCT03449459).

## 3 Results

### 3.1 Demographic characteristics of subjects

**Figure 1:**
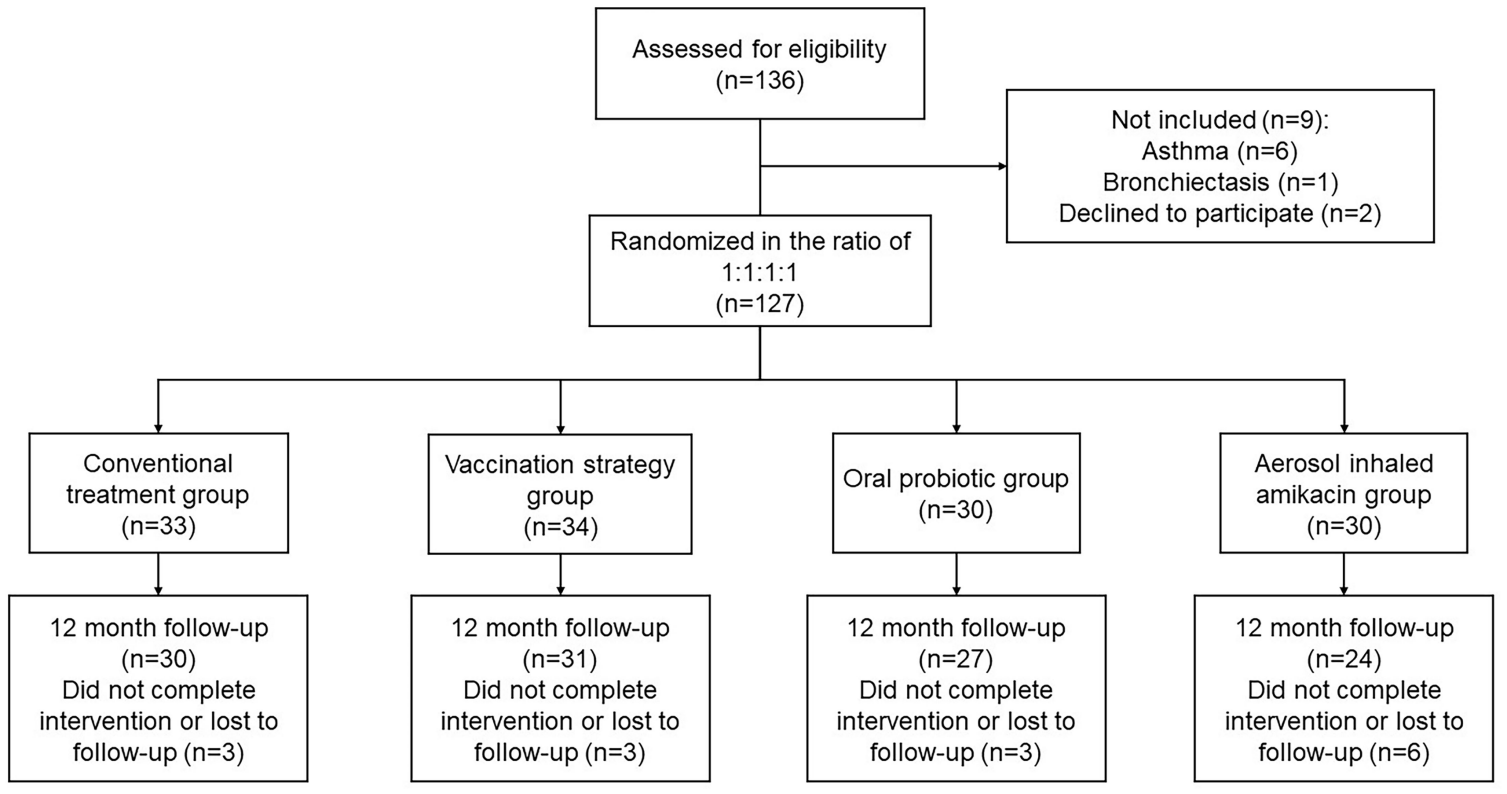
Flow of patients through the study.

In this study, a total of 136 subjects were included in the screening, of which 9 failed due to detection of comorbidities or unwillingness to sign informed consent. One hundred and twenty-seven subjects who met all inclusion criteria and with no exclusion criteria were enrolled in the study. Among all enrolled subjects, there were 15 cases of loss that did not complete the intervention. Therefore, a total of 112 subjects completing 12 months follow-up visits were finally included in the analysis (Figure 1).

Among all 112 analyzed subjects, the average age was 67.19 ± 7.39 years. There were 101 (90.2%) males and 96 (85.7%) patients with a history of smoking (35.18 ± 27.81 pack-years on average). The subjects’ average FEV_1_ was 1.14 ± 0.45 L, the average FEV_1_/predicted FEV_1_ (FEV_1_%) was 41.06 ± 16.09%, and the average FEV_1_/FVC was 50.54 ± 15.57. The demographic characteristics and baseline data of the conventional treatment group, aerosol inhaled amikacin group, oral probiotics group, and vaccination strategy group are shown in Table 1. There was no significant difference within all groups (*p* < 0.05).

**Table 1:** Demographic characteristics and baseline data of subjects.

	The conventional treatment group (*n* = 30)	The vaccination strategy group (*n* = 31)	The oral probiotics group (*n* = 27)	The aerosol inhaled amikacin group (*n* = 24)	*p* value*
Age (years)	66.03 ± 9.23	68.35 ± 6.83	66.04 ± 6.71	68.42 ± 6.12	0.424
Sex, male (%)	26 (86.7%)	27 (87.1%)	26 (96.3%)	22 (91.2%)	0.583
Current or former smokers (%)	22 (73.3%)	27 (87.1%)	26 (96.3%)	21 (87.5%)	0.096
Smoking dose (pack-years)	35.42 ± 36.58	28.97 ± 17.48	42.81 ± 29.11	34.32 ± 24.25	0.313
BMI	22.72 ± 2.95	22.79 ± 3.00	22.87 ± 2.32	22.64 ± 3.18	0.993
FEV_1_ (L)	1.17 ± 0.54	1.11 ± 0.45	1.20 ± 0.38	1.07 ± 0.40	0.726
FEV_1_, %predicted	44.20 ± 17.83	39.13 ± 16.34	42.95 ± 16.41	37.48 ± 12.72	0.376
FEV_1_/FVC	50.24 ± 13.29	50.64 ± 15.40	51.20 ± 16.16	50.05 ± 17.65	0.994
CAT	19.32 ± 9.13	19.95 ± 6.08	20.31 ± 7.14	21.71 ± 4.46	0.753
mMRC ≥ 2 (%)	23 (76.7%)	21 (67.7%)	22 (81.5%)	17 (70.8%)	0.272
Patients with severe or very severe COPD (%)	23 (76.7%)	23 (74.2%)	19 (70.4%)	18 (75.0%)	0.496
Patients with high symptom burden (%)	24 (80.0%)	29 (93.5%)	25 (92.6%)	23 (95.8%)	0.175
Patients with high risk of AE (%)	22 (73.3%)	20 (64.5%)	17 (63.0%)	17 (70.8%)	0.810
Patients labeled GOLD D (%)	19 (63.3%)	18 (58.1%)	16 (59.3%)	16 (66.7%)	0.439
the frequency of moderate-to-severe exacerbations during the year before enrollment	1.63 ± 0.83	1.95 ± 2.04	1.13 ± 0.34	1.43 ± 0.65	0.248

Data are shown as means ± standard deviation or number (%) subjects. *p* value was derived from comparison among groups. *p value < 0.05.

AE, acute exacerbation; BMI, Body Mass Index; COPD, chronic obstructive pulmonary disease; FEV_1_, forced expiratory volume in 1 s; FEV_1_%, FEV_1_/predicted FEV_1_; FEV_1_/FVC, forced expiratory volume in 1 s/forced vital capacity; GOLD, Global initiative for Chronic Obstructive Lung Disease; CAT, COPD Assessment Test; mMRC, Medical Research Council Dyspnea Scale.

### 3.2 Time to the first moderate-to-severe AECOPD

**Figure 2:**
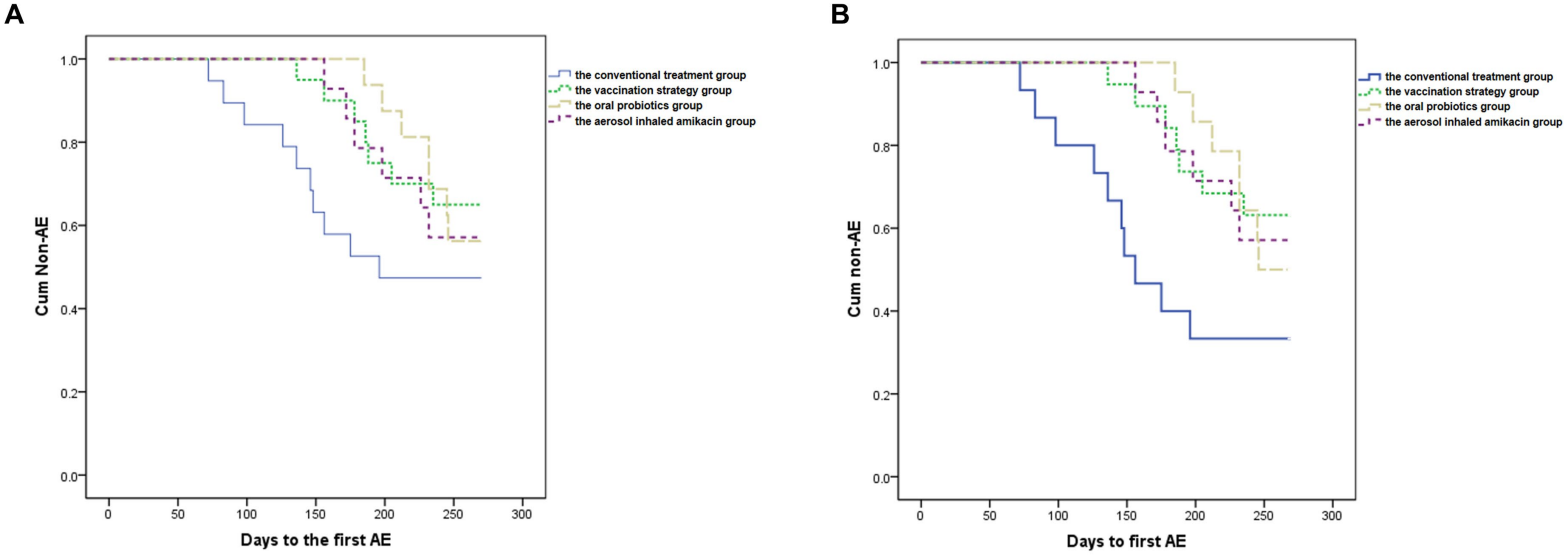
Kaplan–Meier analysis. **(A)** Shows the delay from enrollment to the first moderate-to-severe AECOPD caused by the interventions compared to the conventional treatment in all subjects. *p* = 0.378. **(B)** Shows the significant delay from enrollment to the first moderate-to-severe AECOPD caused by the interventions compared to the conventional treatment in subjects with high symptom burden. *p* = 0.046.

As shown in Table 2 and Figure 2A, ANOVA showed that subjects in all intervention groups took significantly longer days from enrollment to the first moderate-to-severe AECOPD than the conventional treatment group (*p* = 0.026, *F* = 3.307) (Dunnett’s *t* test: the vaccination strategy group, 239.7 vs. 198.2 days, *p* = 0.044, 95%CI [0.85, 82.13]; the oral probiotics group, 248.8 vs. 198.2 days, *p* = 0.017, 95%CI [7.49, 93.59]; the aerosol inhaled amikacin group, 237.3 vs. 198.2 days, *p* = 0.100, 95%CI [−5.61, 83.76]). In addition, the self-control paired *t* test showed that the frequency of moderate-to-severe exacerbations of subjects during the follow-up period was significantly lower than that during the year before enrollment in each group (the conventional treatment group: 0.84 vs. 1.63 per year, *p* = 0.000, 95%CI [0.43, 1.16]; the vaccination strategy group: 0.53 vs. 1.95 per year, *p* = 0.002, 95%CI [0.58, 2.26]; the oral probiotics group: 0.58 vs. 1.13 per year, *p* = 0.011, 95%CI [0.15, 0.94]; the aerosol inhaled amikacin group: 0.57 vs. 1.43 per year, *p* = 0.000, 95%CI [0.50, 1.22]).

**Table 2:** The occurrence of moderate-to-severe AECOPD in the follow-up period.

	The conventional treatment group (*n* = 30)	The vaccination strategy group (*n* = 31)	The oral probiotics group (*n* = 27)	The aerosol inhaled amikacin group (*n* = 24)	*p* value*
days to the first moderate-to-severe AECOPD	198.2 ± 75.4	239.7 ± 46.5	248.8 ± 29.0	237.3 ± 43.5	0.026*
the frequency of moderate-to-severe exacerbations	0.84 ± 0.91	0.53 ± 0.79	0.58 ± 0.68	0.57 ± 0.68	0.614
the difference of the frequency of moderate-to-severe AE before and after the treatment	0.79 ± 0.75	1.42 ± 1.79	0.54 ± 0.75	0.86 ± 0.62	0.130

Data are shown as means ± standard deviation. *p* value was derived from comparison among groups. *p value < 0.05.

AECOPD, acute exacerbation of chronic obstructive pulmonary disease; AE, acute exacerbation.

Meanwhile, we performed the subgroup analysis in terms of whether subjects were severely or very severely airflow limited, whether subjects had a high risk of exacerbation, whether subjects had a high symptom burden or whether subjects were labeled GOLD D. ANOVA demonstrated that in the subgroup of subjects with high symptom burden, all three intervention significantly delayed the exacerbation in contrast with the conventional treatment group (*p* = 0.002, *F* = 5.482) (Dunnett’s *t* test: the vaccination strategy group, 238.1 vs. 179.1 days, *p* = 0.004, 95%CI [16.45, 101.62]; the oral probiotics group, 245.7 vs. 179.1 days, *p* = 0.003, 95%CI [20.83, 112.47]; the aerosol inhaled amikacin group, 237.3 vs. 179.1 days, *p* = 0.009, 95%CI [12.40, 104.04]), which was also proven by Kaplan–Meier analysis (see Figure 2B).

### 3.3 Secondary endpoints

#### 3.3.1 Cat score

**Figure 3:**
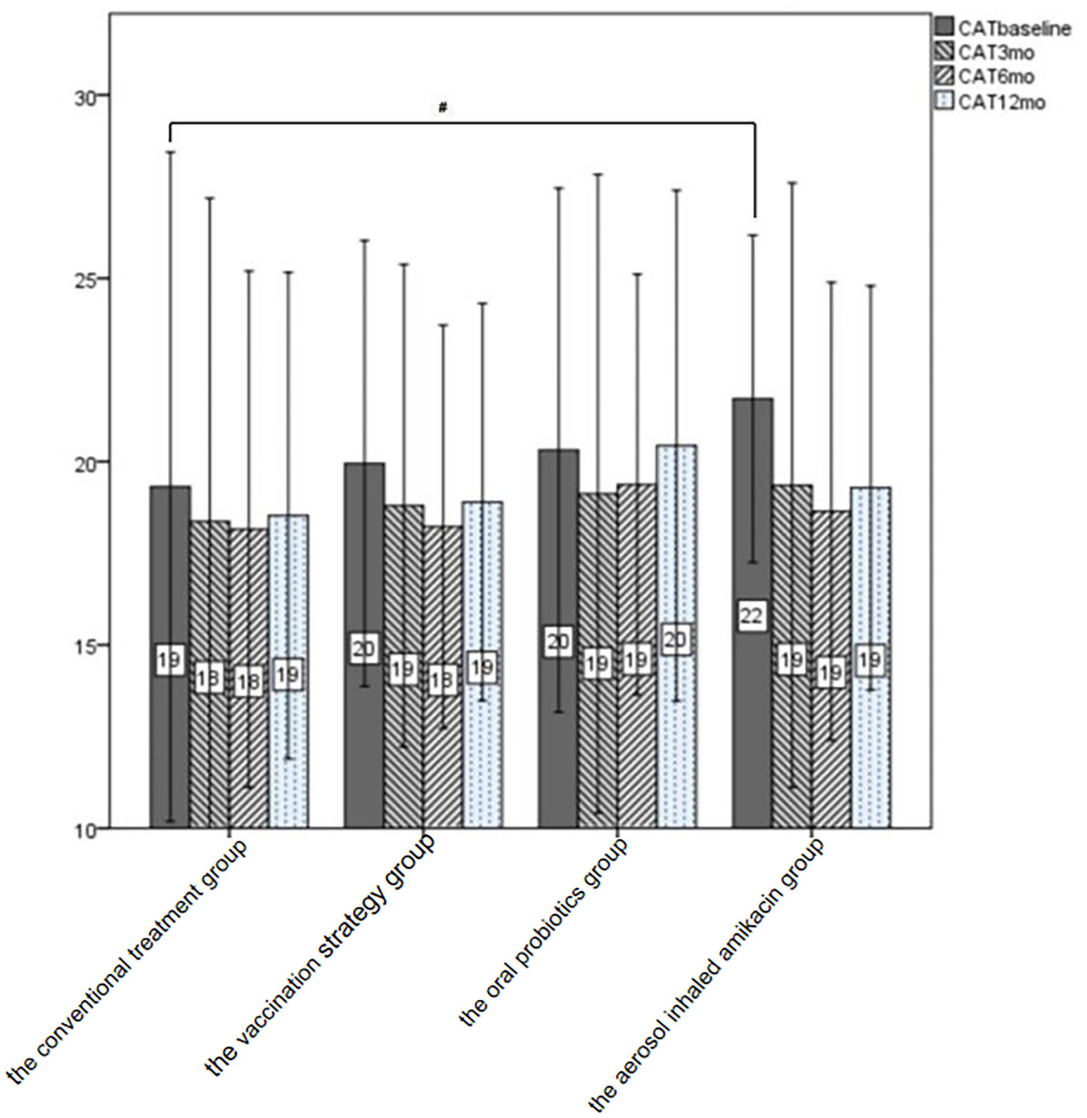
CAT of all subjects at baseline and follow-up. After treatment, the CAT score at each follow-up was lower than that at the baseline visit in all the intervention groups and the conventional treatment group. ^#^*p* < 0.05.

As shown in Table 3 and Figure 3, we could not consider that any of the interventions in this study significantly improved the subject’s CAT score at each follow-up visit by means of the analysis of variance. In addition, we performed the subgroup analysis in terms of whether subjects were severely or very severely airflow limited, whether subjects had a high risk of exacerbation, whether subjects had a high symptom burden or whether subjects were labeled GOLD D. The self-control paired *t*-test showed that in subjects labeled GOLD D, those who were given oral probiotic had a significant improvement in the CAT score by 2.2 at the end of follow-up compared with the baseline (*p* = 0.02, 95%CI [0.6, 3.8]). And in subjects with high symptom burden, those who were given dual vaccine and aerosol inhaled amikacin had a significant improvement in the CAT score by 2.75 (*p* = 0.029, 95%CI [0.3, 5.1]) and 3.07 (*p* = 0.045, 95%CI [0.38, 6.2]), respectively.

**Table 3:** CAT of all subjects at baseline and follow-up visits.

	The conventional treatment group (*n* = 30)	The vaccination strategy group (*n* = 31)	The oral probiotics group (n = 27)	The aerosol inhaled amikacin group (n = 24)	*p* value*
Baseline	19.32 ± 9.13	19.95 ± 6.08	20.31 ± 7.14	21.71 ± 4.46	0.753
3-month visit	18.37 ± 8.82	18.8 ± 6.58	19.13 ± 8.71	19.36 ± 8.24	0.986
6-month visit	18.16 ± 7.04	18.23 ± 5.5	19.38 ± 5.74	18.64 ± 6.25^#^	0.935
12-month visit	18.53 ± 6.64	18.9 ± 5.42	20.44 ± 7.0	19.29 ± 6.10	0.821

Data are shown as means ± standard deviation. *p value was derived from comparison among groups. ^#^*p* value < 0.05 (self-control paired *t* test).

#### 3.3.2 mMRC score

As shown in Table 4, we could not consider that any of the interventions in this study significantly improved the subject’s mMRC score at each follow-up visit by means of the analysis of variance. After intervention, the mMRC scores of the subjects in all groups has improved at each follow-up visit compared with the baseline, but the improvement was not statistically significant through self-control paired *t* test.

**Table 4:** The proportion of mMRC ≥2 in all subjects at baseline and follow-up visits.

	The conventional treatment group (*n* = 30)	The vaccination strategy group (*n* = 31)	The oral probiotics group (*n* = 27)	The aerosol inhaled amikacin group (*n* = 24)	*p* value*
Baseline	23 (76.7%)	21 (67.7%)	22 (81.5%)	17 (70.8%)	0.272
3-month visit	22 (73.7%)	22 (70.0%)	20 (75.0%)	14 (57.1%)	0.700
6-month visit	19 (63.2%)	19 (60.0%)	20 (75.0%)	15 (64.3%)	0.499
12-month visit	20 (68.4%)	19 (60.0%)	20 (75.0%)	17 (71.4%)	0.613

Data are shown as number (%) subjects. *p* value was derived from comparison among groups. *p value < 0.05.

#### 3.3.3 Adverse effects

There were no deaths during the 12-month follow-up period. Five subjects (20.8%) given aerosol inhaled amikacin developed cough, which could be tolerated after symptomatic treatment. There was no significant change in liver and kidney function in all subjects after intervention.

## 4 Discussion

This pilot study sought to determine whether modulating respiratory microbiota during stable stage of COPD could prevent acute exacerbation. As multiple studies have confirmed (41–43), we found that conventional treatment in accordance with the GOLD 2019 report, including standardized drug treatment and tobacco cessation support, with or without the other three interventions vaccination could significantly decrease the frequency of moderate-to-severe AECOPD. All the four groups had reduced CAT scores and mMRC scores at the 6-month follow-up, but the differences were not all significant. More importantly, the additional administration of influenza-*S. pneumoniae* vaccinations and long-term oral probiotic LGG, respectively, postponed the next onset of AECOPD by 41.5 days (*p* = 0.044) and 50.6 days (*p* = 0.017). The aerosol inhaled amikacin showed the same tendency to delay the exacerbation COPD, but no statistically significant difference was detected (*p* = 0.100), perhaps due to the fact that not all subjects enrolled in this group had high bacterial burden in lower respiratory tract.

The frequency of AECOPD in subjects who were given the influenza-*S. pneumoniae* vaccination decreased the most during the follow-up period compared to the year before enrollment (1.42 ± 1.79 per year, *p* = 0.002), suggesting that dual vaccination might be more effective in preventing AECOPD than the other interventions. We speculated that both the oral probiotics and the aerosol inhaled amikacin lasted only 3 months, whereas the validity period of vaccination could usually be maintained for more than 1 year covering the entire follow-up period, thus making it more advantageous in this study. It remains to be verified whether prolonging the intervention time of the oral probiotics and the aerosol inhaled amikacin help enhance their efficacy. Moreover, it has been reported that patients with influenza infection may be more susceptible to infections of other pathogens, such as *S. pneumococcal,* the mechanism of which is thought to be related to the extensive respiratory epithelial damage caused by the direct effects of the virus, the effects of induced interferon and the actions of cytotoxic T-cells after influenza virus infection (44). Compared with receiving influenza vaccine or *S. pneumococcal* vaccine alone, the concomitant injection of both showed additive effects in reducing the incidence of pneumonia, all-cause mortality, all-cause hospitalizations and inpatient expenditures of all diseases among the elderly (45, 46). Our study demonstrated the significant benefit of influenza-*S. pneumococcal* vaccination in reducing the frequency of AECOPD. Nevertheless, the additive effect of dual influenza and *S. pneumococcal* vaccination compared with separate administration in AECOPD prevention needs to be further verified, which will help to clarify the optimal vaccination mode in stable COPD.

Previous studies have found the crucial bidirectional connection between the intestinal microbiota and the lungs, namely the “Gut-Lung axis” (47). In respiratory infection diseases, modulating gut microbiota by oral probiotics could reduce the duration of intensive care units (ICU) admission, the severity of the common cold and the incidence of ventilator-associated pneumonia (VAP) and upper respiratory infections (48). As for chronic respiratory diseases, oral probiotics could significantly improve allergic rhinitis via improving at clinical signs, decreasing the rate of exacerbation, and reducing the use of relieving medication (24, 49). However, despite improving serum inflammatory factors and cytokines, oral probiotics could not significantly improve signs and symptoms in asthma (50). Although a reduction in alveolar inflammatory cells infiltration and subsequent lung damage was observed in emphysema mice receiving oral probiotics (51), studies evaluating the clinical effects of oral probiotics on COPD are still limited. This is the first clinical trial to report that oral probiotic LGG can delay the occurrence of AECOPD, reduce the frequency of AECOPD, and improve symptoms in patients labeled GOLD D, suggesting that COPD is also a chronic respiratory disease that can benefit from intestinal microbiota regulation. Better understanding of the “Gut-Lung axis” is needed to design gut microbiota-associated strategies for the treatment and prevention of COPD.

Results from the few previous studies of long-term inhaled antibiotics in patients with stable COPD have been discouraging. A clinical trial (NCT00739648) conducted in the United States observed no changes in exacerbation rate after levofloxacin inhalation. Bruguera-Avila N and colleagues found that long-term inhalation of colistin for at least 3 months was not associated with the number of AECOPD cases not requiring admission in COPD patients with bronchial colonization by *Pseudomonas aeruginosa* but could decrease the hospitalization and the length of hospital stay (52). These results suggested the necessity of selecting sensitive antibiotics and the possibility that inhaled antibiotics may be more effective in preventing severer AECOPD. Most of the stable COPD patients have airway bacterial colonization dominated by Gran-negative bacteria, including *Moraxella pneumoniae*, *S. pneumoniae* and *P. aeruginosa* (53), which can be covered by amikacin. Our study found that inhaled amikacin did not significantly delay the occurrence of AECOPD. However, in the subgroup analysis, patients with high symptom burden had prolonged onset of moderate-to-severe AECOPD after receiving any of the three interventions, including aerosol amikacin (237.3 vs. 198.2 days), compared to the conventional group. Additionally, only the amikacin group had a significant improvement in CAT scores at the 6-month follow-up (18.64 vs. 21.71). It suggests that patients with high symptom burden may represent a group of people with specific airway microbiota and high load of respiratory tract colonization. These patients may be more likely to benefit from appropriate inhaled antibiotics. Thus, the inclusion criteria of future studies on the nebulized antibiotics against respiratory decolonization should emphasize the isolation of bacteria (such as *P. aeruginosa*) or the manifestations of bronchitis (such as cough and sputum).

The COVID-19 pandemic has become a major obstacle to conducting this study, thus resulting in several limitations. Due to the social policies for epidemic control, part of the follow-up was completed through telephone visits, which may affect the accuracy of the outcome assessment. However, the onset of moderate-to-severe AECOPD is a relatively objective event according to the criteria defined in this study, so we believe that the primary outcome data collected are reliable. Without the onsite visit, we were unable to collect blood and sputum samples, which made it impossible to complete the planned microbial study and restricted the analysis of existing clinical indicators. In addition, the community epidemic prevention measures, such as wearing a mask, carried out in Shanghai and Guangzhou since the beginning of 2020 resulted in a visual reduction in respiratory infections (e.g., flu) and AECOPD, which could reduce the effects of the three interventions.

## 5 Conclusion

For moderate-to-very severe COPD patients with a history of moderate-to-severe exacerbations, the combined vaccination (influenza and *Streptococcus pneumoniae* vaccine) and continuous oral probiotic LGG for 3 months can significantly delay the next moderate-to-severe exacerbation. Similarly, for patients with high symptom burden, the aerosol inhaled amikacin also significantly delayed the next moderate-to-severe exacerbation.

## Data availability statement

The raw data supporting the conclusions of this article will be made available by the authors, without undue reservation.

## Ethics statement

The trial has been approved in the Ethics Committee of Zhongshan Hospital of Fudan University (B2017-197R). The studies were conducted in accordance with the local legislation and institutional requirements. The participants provided their written informed consent to participate in this study.

## Author contributions

J-lH: Conceptualization, Data curation, Formal analysis, Funding acquisition, Investigation, Methodology, Project administration, Resources, Visualization, Writing – original draft, Writing – review & editing. Z-fY: Conceptualization, Data curation, Formal analysis, Funding acquisition, Investigation, Methodology, Project administration, Resources, Writing – review & editing. Q-jC: Conceptualization, Data curation, Formal analysis, Funding acquisition, Investigation, Methodology, Writing – review & editing. Y-pH: Conceptualization, Data curation, Formal analysis, Funding acquisition, Investigation, Methodology, Validation, Writing – review & editing. Z-tL: Data curation, Investigation, Project administration, Writing – review & editing. R-rD: Data curation, Investigation, Project administration, Writing – review & editing. B-fH: Formal analysis, Investigation, Visualization, Writing – review & editing. Y-xW: Formal analysis, Investigation, Software, Validation, Writing – review & editing. JZ: Conceptualization, Data curation, Formal analysis, Funding acquisition, Investigation, Methodology, Project administration, Resources, Supervision, Visualization, Writing – original draft, Writing – review & editing.
